# Microenvironment Responsive Modulations in the Fatty Acid Content, Cell Surface Hydrophobicity, and Adhesion of *Candida albicans* Cells

**DOI:** 10.3390/jof4020047

**Published:** 2018-04-06

**Authors:** Asha Bhujangrao Shiradhone, Sujata S. Ingle, Gajanan B. Zore

**Affiliations:** Research Laboratory 1, School of Life Sciences, Swami Ramanand Teerth Marathwada University, Nanded431606, MS, India; manasvisuhani@yahoo.com (A.B.S.); inglesujata9@yahoo.com (S.S.I.)

**Keywords:** cell surface hydrophobicity, adhesion, ergosterol, fatty acids, lipids, membrane fluidity

## Abstract

Considering the significance in survival and virulence, we have made an attempt to understand modulations in the membrane and cell wall properties of *Candida albicans* hyphae induced by temperature (37 °C) and neutral pH and yeast form cells grown under low hydrostatic pressure (LHP). Our results suggest that cell surface hydrophobicity (CSH) and adhesion are dynamic properties determined largely by the microenvironment rather than morphological forms, citing the significance of variation in niche specific virulence. GC-MS analysis showed that 49 and 41 fatty acids modulated under hyphal form induced by temperature alone (37 °C) and neutral pH, respectively while that of 58 under yeast form cells under low hydrostatic pressure (LHP) (1800 Pa). Fatty acid and ergosterol data indicates that fluidity increases with increase in temperature (37 °C) and neutral pH i.e., saturated fatty acids and ergosterol decreases. Similarly, CSH and adhesion decrease in response to temperature (37 °C), pH 7, and LHP compared to controls, irrespective of morphological forms. In general, membranes were more rigid, and cell walls were more hydrophobic and adhesive in yeast form compared to hyphal form cells, except in case of yeast form cells grown under LHP. Yeast form cells grown under LHP are less hydrophobic and adhesive.

## 1. Introduction

*Candida albicans* is one of the most frequent opportunistic pathogens that establish difficult-to-treat invasive candidiasis, including bloodstream infections (Candidemia) and biofilms, upon the immunocompromised condition and/or imbalance in body-micro flora [1,2,3]. *Candida* species are the fourth most common cause of nosocomial bloodstream infection, with a crude mortality rate of 50%, the highest amongst nosocomial bloodstream infections [4,5]. The extended stay in intensive care units (ICU), immunocompromised condition, use of intravenous catheters, total parenteral nutrition, invasive procedures, and the increasing use of broad-spectrum antibiotics, cytotoxic chemotherapies, and transplantation are important predisposing factors for *C. albicans* infections [2,4,6].

*C. albicans* is a polymorphic fungus that exists in the form of yeast, pseudohyphae, hyphae, chlamydospores, and opaque cells [7,8]. Its ability to change morphology makes *C. albicans* one of the most successful opportunistic pathogens of humans that can infect almost all the tissue sites with different and extreme micro-environments [1,7,9]. In addition to this, cells in different morphological forms often exhibit differential responses toward host defense and/or anti-fungal agents leading to the emergence of drug resistance and thus survival. Bloodstream infections lead colonization of medical devices in the form of drug-resistant biofilms (different morphological forms of *C. albicans* cells as well as cells in biofilms, are inaccessible to defense and or anti-fungal agents) [10]. The hyphalform is essential for invading host tissues, and hyphae and opaque cells evade host immune responses [11].

Membrane and cell wall properties, though implicated in morphogenesis and thus virulence of *C. albicans*, are hypothesized to be modulated due to environmental or nutritional factors rather than morphological forms [12]. *C. albicans* membranes are rich in sterols (1.2%) and phospholipids (1.1%), and fatty acids decide the membrane properties like fluidity, signaling, and transport and thus significantly modulate virulence and drug resistance [13,14,15]. Membrane fluidity and lipids content are linked to the multidrug drug resistance in *C. albicans*, especially against azole, allylamine, and polyene antifungal agents in addition to adaptation to extreme microenvironments [16,17,18].

Its ability to form hyphae and hyphae specific cell surface molecules (Adhesin, SAPs, Lip, PL, etc.) are the major virulence factors of *C. albicans* [1,19,20,21]. Cell surface chemistry (cell surface hydrophobicity and adhesion) plays an important role in the pathogenicity of *C. albicans* i.e., cell surface hydrophobicity is reported to be positively correlated with adherence [1,22,23]. In addition to cell surface chemistry, the ability to modulate membrane fluidity is also important in survival and thus virulence in *C. albicans* [24,25]. Elevated temperature (37 °C) and neutral pH induce hyphae, while low hydrostatic pressure (LHP) inhibits hyphae induction in *C. albicans* [9,26]. Interestingly, LHP equivalent to capillary hydrostatic pressure, i.e., 1800 Pa (13mmHg), is enough to inhibit hyphae induction in *C. albicans* [26]. This could be one of the reasons for yeast form growth of *C. albicans* cells during candidemia [27,28,29]. These physical factors include temperature (37 °C), neutral pH, and LHP are present in the host tissue micro-environments. Thus, to understand the microenvironment specific modulations, we have evaluated membrane (fatty acid and ergosterol content) and cell wall (CSH and adhesion) properties under different physical factors.

## 2. Materials and Methods

A standard strain of *Candida albicans* ATCC 10231 was obtained from the Microbial Type Culture Collection (MTCC), Institute of Microbial Technology (IMTECH), Chandigarh, India. The strain was cultured on YEPD (1% yeast extract, 2% peptone, 2% dextrose, and 2.5% agar) agar slants with pH 6.5 and maintained at 4 °C [30]. PBS (Phosphate buffered saline), and all the media components were procured from Hi Media Laboratories Ltd., (Mumbai, India). N-Octane (99.9% pure), n-heptane was purchased from Sigma Aldrich Ltd., Mumbai, India.

### 2.1. Inoculum Preparation

*C. albicans* (ATCC 10231) yeast phase cells grown on YEPD broth for 24h at 28 ± 2 °C were harvested by centrifugation (1000 rpm for 2 min), cells were washed three times with sterile distilled water, re-suspended in 1 mL distilled water and incubated for 1 h at 28 ± 2 °C for starvation. The cell density of starved cells was determined microscopically using hemocytometer and adjusted to 2 × 10^6^ cells/mL. These cells were used in the further study [1,31].

### 2.2. Effect of Physical Factors (pH, Temperature and Low Hydrostatic Pressure) on Fatty Acid Content in Candida albicans

To evaluate the effect of temperature and neutral pH, starved cells (2 × 10^6^ cells/mL) were inoculated into the flasks containing YPD broth of pH 6.5 and pH 7 respectively and incubated at 30 °C and 37 °C. Three flasks/bumper tubes were used for each treatment, and these three samples were combined and processed as a single sample after incubation. Impact of low hydrostatic pressure (LHP) (1800 Pa) on *C. albicans* morphogenesis was evaluated by inoculating starved cells (2 × 10^6^ cells/mL) in 75 mL YPD broth with proline (2.5 mM) in bumper tubes (25 × 200 mm), allowed cells to settle down incubated at 37 °C and compared with control (cells were grown in YPD with 2.5 mM proline at hydrostatic pressure 100 Pa). LHP was calculated by using the formula, P = hpg (where, P—hydrostatic pressure, h—height of the liquid column, p—density of liquid, and g—gravitational constant (9.81 m/s^2^)). All the flasks and bumper tubes were incubated for 6h. Cells were harvested using a centrifuge (1000 rpm for 2 min) and used for fatty acid analysis. The experiment was carried out in triplicate. Cells from each replicate was used for preparing methyl esters of fatty acids [32,33].

### 2.3. Fatty Acid Analysis Using GC-MS

Fatty acids from the *C. albicans* cells grown under different conditions as mentioned above were extracted as per the protocol given by Sasser, M. (1990) [34]. The cell sample of each replicate was processed separately. In brief, loop full cells (40 mg) were added in a tube containing 1 mL of saponification reagent (45 g sodium hydroxide, 150 mL methanol, and 150 mL distilled water), and saponification of lipids was carried out by incubating in a boiling water bath for 30 min with intermittent vortexing. The methylation of fatty acid was carried out by adding 2 mL of methylation reagent (325 mL certified 6.0 N hydrochloric acid and 275 mL methyl alcohol) and incubating for 10 min at 80 °C. After methylation, fatty acids were extracted by adding 1.25 mL of extraction reagent (200 mL hexane and 200 mL methyl tert-butyl ether) and discarding the aqueous (lower) phase. The remaining extract was washed with 3 mL of washing reagent (10.8 g sodium hydroxide dissolved in 900 mL distilled water) and 2/3 of the organic phase (fatty acid methyl esters) was transferred into a GC vial. An equal volume of fatty acid methyl esters from each replicate (three replicates) was constituted in a single vial and analyzed as a single sample using GC-MS for each growth condition.

Samples were analyzed using a 25 m × 0.2 mm phenyl methyl silicone–fused silica capillary column with less noise and drift during temperature programmed runs in Sherlock RTSBA6 Series gas chromatograph. The temperature program ramps from 170 °C to 270 °C at 5 °C per min, and hydrogen was used as carrier gas, nitrogen as makeup gas, and air to support the flame. The electronic signal from the GC detector is provided to the computer to integrate the peaks. The results obtained were compared with the databases using the Sherlock pattern recognition software to analyze fatty acid methyl ester composition of the sample [34].

### 2.4. Identification of Fatty Acids Using Equivalent Chain Length (ECL) Value

External calibration standards (a mixture of the straight chained saturated fatty acids from 9 to 20 carbons in length and five hydroxy acids) developed and manufactured by Microbial ID, Inc. was used to calibrate Equivalent Chain Length (ECL) data for fatty acid identification. The ECL value for each fatty acid can be derived as a function of its elution time in relation to the elution time of a known series of straight chain fatty acids [34]. Short, medium and long chain saturated fatty acids were grouped as per carbon number [34].

ECL_x_= (R_tx_− R_tn_/R_t (n+1)_ –R_tn_) + n

where R_tx_ is the retention time of x, R_tn_ is the retention time of the saturated fatty acid methyl ester preceding x, and R_t(n+1)_ is the retention time of the saturated fatty acid methyl ester eluting after x.

### 2.5. Evaluation of Cell Surface Hydrophobicity (CSH)

Modulation in cell surface hydrophobicity of *C. albicans* cells in response to different microenvironmental factors, including temperature, pH, and LHP, was analyzed as per Hazen and Hazen (1987) and Rosenberg, Gutnick and Rosenberg (1980) [35,36]. In brief, cells grown under different conditions as mentioned above were harvested, washed and resuspended in PBS to yield the optical density of 0.5 at 620 nm. This cell suspension was distributed into three test tubes (1.3 mL each). Afterward, 100 µL from each of these test tubes were distributed in the wells of 96 well microtiter plates and initial OD was read at 620 nm using a Thermoscan-Ex micro plate reader (Thermo Fisher Scientific Inc., 168 3rd Ave, Waltham, MA 02451, USA). Then, 0.3 mL of octane was added to the remaining cell suspension (1.2 mL) in each tube, mixed vigorously for 3 min and allowed to separate for 15 min. 100 µL of the lower aqueous phase was carefully added to the wells of micro titer plate and final O.D was recorded at 620 nm. Tubes without cells were served as control. Triplicates were used for each sample and experiment was repeated thrice. The percentage cell surface hydrophobicity was calculated by using the following formula and results were presented as percentage of CSH ± SD (standard deviation) [35,36].

Percentage CSH = (1 − final OD of aqueous phase/initial OD of cell suspension) × 100.

### 2.6. Evaluation of Adhesion

Impact of environmental factors (Temperature, pH, and LHP) on adhesion of *C. albicans* cells was evaluated as per Panagoda et al. (2001) and He et al. (2005) [37,38]. In brief, 100 µL of the cell suspension (containing 1 × 10^7^ cells/mL) were inoculated into the wells of 96 well flat bottom polystyrene make microtiter plates (Tarson, India), and incubated for 90 min at 37 °C with gentle shaking (50 rpm) on an orbital shaker for adhesion. After incubation, wells were washed three times with PBS to remove un-adhered cells. The number of adhered cells was counted using an inverted research microscope (Metzer, India) and counting the number of cells in each field. Cells from 10 different fields from each well were counted, and the mean value was calculated. Triplicates were used for each sample and experiment was repeated thrice. The number of cells adhering per microscopic field indicated adherence, and results were presented as percentage of adhesion ± SD (standard deviation) [37,38].

### 2.7. Extraction and Estimation of Ergosterol Content

Ergosterol content in the *C. albicans* cells grown under different microenvironmental conditions was estimated as per Arthington-Skaggs et al. (1999) [39]. In brief, cells grown under different micro-environments, including temperature, pH, and LHP (as mentioned above), for 6 h were harvested by centrifugation at 1000 rpm for 2 min. The pellets were washed with sterile distilled water for 2–3 times. The washed cell pellet (0.1 g was suspended into 300 µL of 25% ethanolic KOH (25 g KOH in 35 mL distilled water diluted to 100 mL with ethanol) and incubated at 85 °C for 1 h. Vials were allowed to cool down to room temperature and sterols were extracted using 75% (*v*/*v*) n-heptane (300 µL n-heptane + 100 µL distilled water) with simultaneous vortexing. After vortexing, vials were allowed to separate the layers. The n-heptane layer was transferred carefully into new vial. 200 µL of n-heptane layer was diluted fivefold (100 µL n-heptane + 400 µL ethanol) with 100% of ethanol. The spectrum of the diluted sample was observed at the wavelength ranging from 230–300 nm using a UV-Visible spectrophotometer (Shimadzu Analytical (India) Pvt. Ltd. Mumbai- 400 059, India).

The ergosterol content was determined by using the values of absorbance at 230 nm and 281.5 nm and the following formula [39].

Percentage ergosterol +% 24(28) DHE = [(A281.5/290) × F]/pellet weight,

%24(28) DHE = [(A230/518) × F]/pellet weight,

So, percentage ergosterol = [percentage ergosterol + 24(28) DHE] − %24(28) DHE,

where, F is the factor for dilution in ethanol and 290 and 518 are the E values (in percent per centimeter) determined for crystalline ergosterol and 24(28) DHE, respectively. Results were presented as percentage of ergosterol ± SD (standard deviation).

## 3. Result

### 3.1. Identification of Modulations in Fatty Acid Composition in C. albicans (ATCC10231) Cells in Response to Temperature

**Figure 1:**
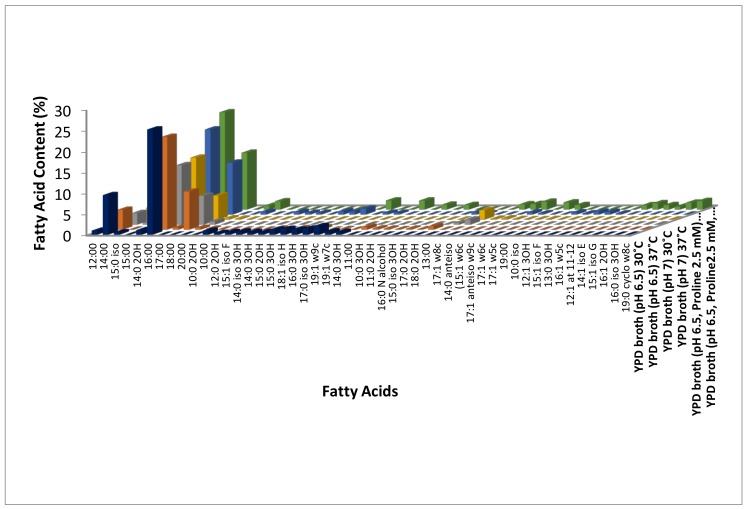
Modulation of saturated fatty acid content in *C. albicans* response to different microenvironments (temperature, pH 7, and hydrostatic pressure).

**Figure 2:**
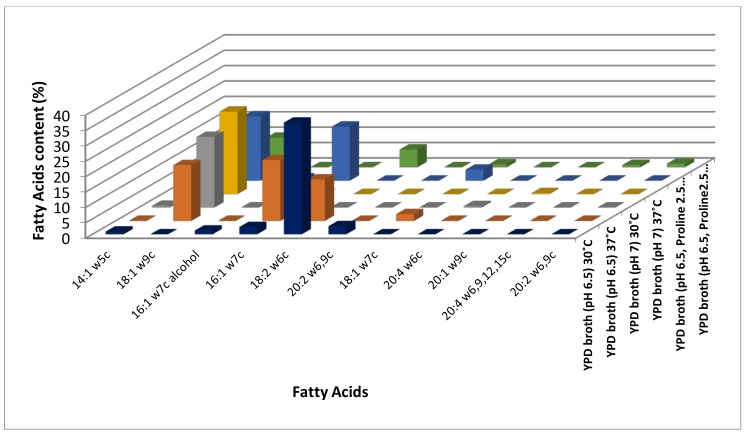
Modulation of unsaturated fatty acids content in *C. albicans* response to different microenvironments (temperature, pH 7, and hydrostatic pressure).

**Figure 3:**
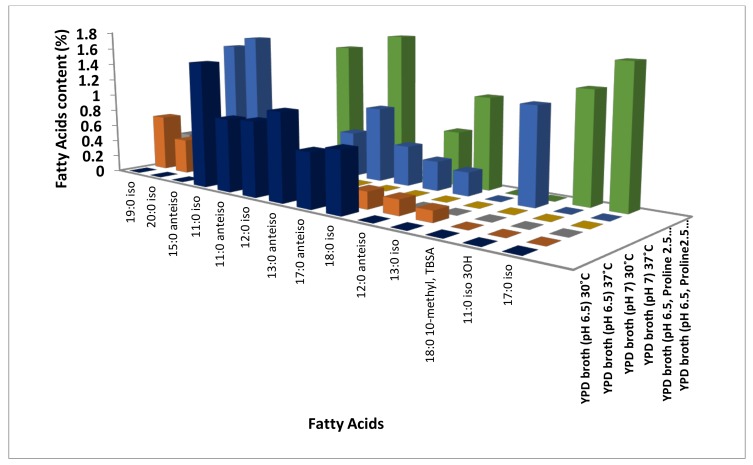
Modulation of methyl branched saturated fatty acids content in *C. albicans* response to different microenvironments (temperature, pH 7, and hydrostatic pressure).

**Figure 4:**
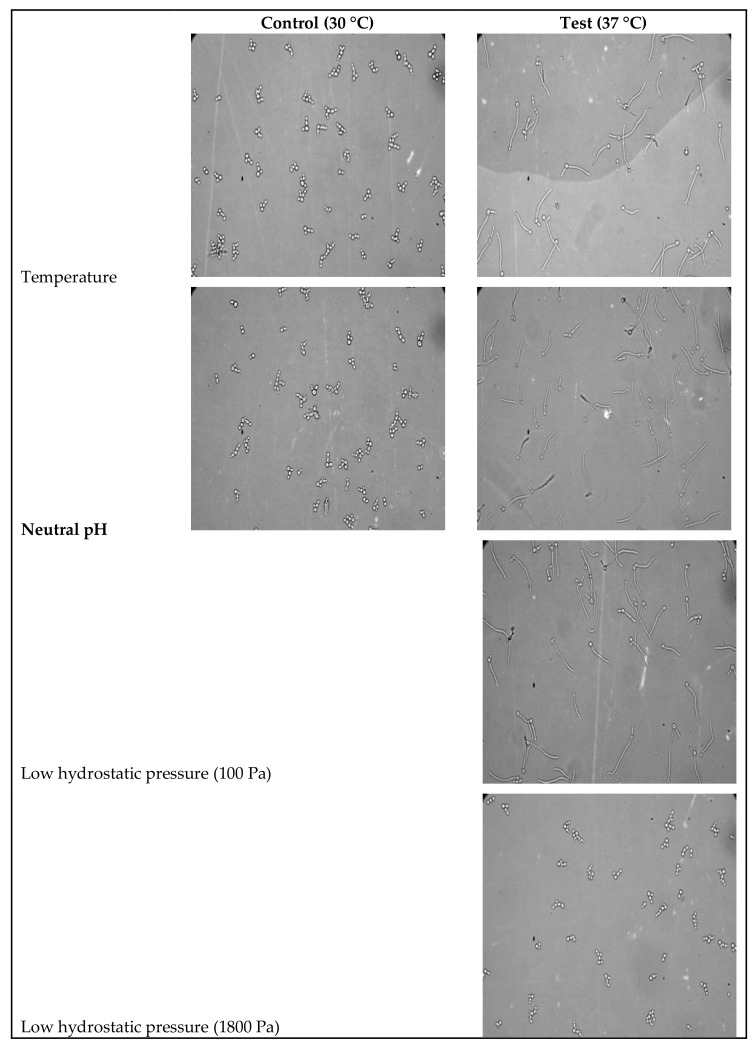
Light microscopy (10×, 40×) images of *C. albicans* yeast and hyphal form cells growing under temperatures (30 °C and 37 °C, neutral pH, and LHP (100 Pa and 1800 Pa)).

Temperature is known to modulate membrane properties like rigidity, fluidity, transport, and signal transduction. It is also known to induce hyphae in *C. albicans* at 37 °C. In the present study, we have identified the fatty acid content of the *C. albicans* cells grown at 30 °C and 37 °C temperature using GC-MS analysis to understand temperature-induced modulations. *C. albicans* induced hyphae at 37 °C (65%) while existing in yeast form at 30 °C (Table 1, Figure 4). A total of 30 fatty acids were identified by Fatty Acid Methyl Ester (FAME) using GC-MS analysis in response to temperature at 30 °C, pH 6.5 (yeast form) while 32 at 37 °C, pH 6.5 (hyphae). Among these, 15 fatty acids were common in both temperatures (i.e., yeast and hyphal form) (Figure 1, Figure 2 and Figure 3, Supplementary Table S1, Figures S1 and S2).

Among these, all the three hydroxyl saturated fatty acids (2-Hydroxytetradecanoic acid, 2-Hydroxydodecanoic acid, and 3-Hydroxy-12-Methyltridecanoic acid) identified were up-regulated at 30 °C (Supplementary Table S1, Figure 1, Figures S1 and S2). Out of the 20 long-chain saturated fatty acids, 13and seven were up-regulated at 30 °C and 37 °C, respectively (Figure 1), while two (Dodecanoic acid and Decanoic acid) and three medium-chain saturated fatty acids (Undecanoic acid, 3-Hydroxydecanoic acid and 11:0 2OH) were up-regulated at 30 °C and 37 °C, respectively (Supplementary Table S1, Figure 1, Figures S1 and S2). Out of the 14 methyl-branched fatty acids identified, six (9-Methyldecanoic acid, 8-Methyldecanoic acid, 10-Methylundecanoic acid, 10-Methyldodecanoic acid, 14-Methylhexadecanoic acid, and 16-Methylheptadecanoic acid) were up-regulated at 30 °C, and five (17-Methyloctadecanoic acid, 18-Methylnonadecanoic acid, 12-Methyltetradecanoic acid, 9-Methylundecanoic acid and 11-Methyldodecanoic acid) at 37 °C (Supplementary Table S1, Figure 3, Figures S1 and S2). In general, most of the saturated fatty acids were abundant at 30 °C (Supplementary Table S1, Figure 1, Figures S1 and S2). Surprisingly, four (9Z)-9-Tetradecenoic acid, (9Z)-9-Hexadecen-1-ol, (9Z,12Z)-9,12-Octadecadienoic acid and (11Z,14Z)-11,14-Icosadienoic acid) out of the five unsaturated fatty acids and a short chain fatty acid (2-Hydroxydecanoic acid) was up-regulated at 30 °C (Supplementary Table S1, Figure 2). One ((11Z)-11-Octadecenoic acid), two monounsaturated fatty acids (Monounsaturated and Omega-7 monounsaturated), and a short-chain saturated (11Z)-11-Octadecenoic acid) fatty acid were up-regulated 37 °C (Supplementary Table S1, Figure 2, Figures S1 and S2).

### 3.2. Identification of Modulations in Fatty Acid Composition in C. albicans (ATCC10231) Cells in Response to Neutral pH

pH is also one of the most important physical factors, known to induce morphogenesis in *C. albicans* [1,40]. In the present study, we have identified modulation in the fatty acid content of the *C. albicans* cells in response to neutral pH at 30 °C and 37 °C (Supplementary Table S1, Figures S3 and S4) (Figure 4). A total of 41 fatty acids were found to be modulated in response to neutral pH (Supplementary Table S1). Among these, 20 fatty acids found at both, 30 °C and 37 °C were modulated in response to neutral pH (Supplementary Table S1). Thirteen and seven fatty acids were identified at 37 °C (hyphal form) and 30 °C (yeast form), respectively in response to neutral pH (Supplementary Table S1, Figures S3 and S4).

Among the saturated fatty acids identified, one medium chain saturated (Dodecanoic acid), one hydroxy saturated (2-Hydroxytetradecanoic acid), one long chain saturated (13-Methyltetradecanoic acid), and two out of the three methyl-branched saturated fatty acids (17-Methyloctadecanoic acid and 18-Methylnonadecanoic acid) were up-regulated at 30 °C (Supplementary Table S1, Figure 1 and Figure 3, Figures S3 and S4). Thirteen out of the 14 long-chain saturated fatty acids and one methyl branched saturated (12-Methyltetradecanoic acid) fatty acid were up regulated at 37 °C in response to neutral pH (Supplementary Table S1, Figure 1 and Figure 3, Figures S3 and S4). Both the monounsaturated fatty acids (Monounsaturated and Monounsaturated Omega-9) identified and one among the two ((9Z)-9-Tetradecenoic acid) unsaturated fatty acid were up-regulated at 37 °C (Supplementary Table S1, Figure 2, Figures S3 and S4). In general, saturated fatty acids, except long-chain fatty acids, were up-regulated at 30 °C, while unsaturated fatty acids were up-regulated at 37 °C in response to neutral pH (Supplementary Table S1, Figure 1 and Figure 2, Figures S3 and S4).

### 3.3. Identification of Modulations in Fatty Acid Composition in C. albicans (ATCC10231) Cells in Response to LHP (1800 Pa)

The high hydrostatic pressure is one of the most important environmental factors known to modulate fatty acid content in cellular membranes in addition to several other cellular functions [29]. Impact of low hydrostatic pressure (LHP) (1800 Pa) on hyphae induction and fatty acid content of *C. albicans* cells (yeast and hyphae) was evaluated in the present study. LHP (1800 Pa) inhibited hyphae induction by 90% under hyphae inducing condition (Table 1, Figure 4). Fatty acid analysis showed that 58 fatty acids were modulated significantly by LHP in our study (Supplementary Table S1, Figures S5 and S6). Out of these, 12 fatty acids were common in presence of LHP (yeast) and absence of LHP (hyphae) at 37 °C (Supplementary Table S1, Figures S5 and S6). Twenty-five fatty acids were identified only in absence of LHP, i.e., hyphal form, while 21 fatty acids were identified in presence of LHP (yeast) at 37 °C (Supplementary Table S1, Figures S5 and S6).

Four out of the eight medium chain saturated (10:0 iso, 12:1 3OH, 13:0 3OH and 12:1 at 11–12) fatty acids, 13 long chain saturated, five methyl branched saturated (17-Methyloctadecanoic acid, 18-Methylnonadecanoic acid, 10-Methylundecanoic acid, 14-Methylhexadecanoic acid and 10-Methyloctadecanoic acid), and two hydroxyl saturated (2-Hydroxytetradecanoic acid and 3-Hydroxy-12-Methyltridecanoic acid) fatty acids were up regulated in absence of LHP (hyphal form cells) at 37 °C (Supplementary Table S1, Figure 1 and Figure 3, Figures S5 and S6). Fourteen long-chain saturated, four medium-chain saturated (Dodecanoic acid, Decanoicacid, Undecanoic acid and 11:0 2OH), five methyl-branched saturated (8-Methyldecanoic acid, 10-Methyldodecanoic acid, 16-Methylheptadecanoic acid, 9-Methylundecanoic acid and 15-Methylhexadecanoic acid), and one short chain saturated (2-Hydroxydecanoic acid) fatty acid were up regulated in presence of LHP (yeast form cells) at 37 °C (Supplementary Table S1, Figure 1 and Figure 3, Figures S5 and S6). In addition to this, three (Polyunsaturated Omega-6, (5Z,8Z,11Z,14Z)-5,8,11,14-Icosatetraenoic acid and (11Z,14Z)-11,14-Icosadienoic acid) out of five unsaturated fatty acids and one (3-Hydroxy-9-Methyldecanoic acid) branched hydroxyl saturated fatty acid were up-regulated in presence of LHP (yeast form cells) at 37 °C (Figure 2, Figures S5 and S6). Interestingly, monounsaturated fatty acids up-regulated in response to temperature alone (37 °C) and neutral pH at 37 °C (hyphal form cells) were down-regulated in response to LHP (yeast form cells) at 37 °C (Supplementary Table S1, Figure 2, Figures S5 and S6).

### 3.4. Modulation of Cell Surface Hydrophobicity (CSH) and Adhesion

Cell surface hydrophobicity and adhesion are two important virulence factors known to be modulated in response to stress [1]. In our study, physical factors including temperature, pH, and LHP significantly modulated cell surface hydrophobicity and adhesion.

#### 3.4.1. CSH

The modulation of cell surface hydrophobicity of *C. albicans* cells (yeast and hyphae) in response to three environmental factors (temperature, neutral pH, and LHP) was evaluated in this study. CSH of *C. albicans* cells grown in YPD (pH 6.5) at 30 °C (yeast form cells) and at 37 °C (hyphal form cells) was found to be 26.36 ± 1.57% and 22.87 ± 0.5%, respectively (Table 1). CSH of *C. albicans* cells grown in YPD (pH 7) at 30 °C (yeast) and 37 °C (hyphae) was found to be 11.71 ± 0.56% and 6.6 ± 0.48%, respectively (Table 1). CSH of *C. albicans* cells grown in the presence of LHP (YPD pH 6.5 with 2.5 mM Proline and LHP-1800 Pa) (yeast) and the absence of LHP (YPD pH 6.5 with 2.5 mM Proline) (hyphal form cells) was found to be 5.97 ± 0.07% and10.3 ± 0.39%, respectively (Table 1). In general, CSH of *C. albicans* yeast form cells is more compared to hyphal form cells. However, CSH of yeast form cells growing under LHP (1800 Pa) was significantly reduced (5.97 ± 0.07%) compared to even the hyphal form cells growing in absence of LHP (1800 Pa) (Table 1).

#### 3.4.2. Adhesion

Adhesion, one of the most important virulence determinants of the *C. albicans* cells, is reported being modulated by environmental factors [1]. In the present study, we have evaluated modulation of adhesion of *C. albicans* cells in response to temperature, neutral pH and LHP. Adhesion of *C. albicans* cells grown in YPD (pH 6.5) at 30 °C (yeast) and 37 °C (hyphae) to polystyrene was found to be 56 ± 3% and 23.67 ± 2.08%, respectively, while that of in YPD (pH 7) at 30 °C and 37 °C were 68 ± 4% and 30.6 ± 1.52%, respectively (Table 1). On the other hand, adhesion of *C. albicans* cells grown in the presence (yeast form) and absence (hyphal form) of LHP (1800 Pa) was found to be 20.66 ± 1.52% and 51 ± 1%, respectively (Table 1).

### 3.5. Ergosterol

The spectrophotometric analysis showed that ergosterol content was more in yeast phase cells of *C. albicans* compared to hyphae (Table 1). Percent ergosterol content of yeast phase cells grown in YPD (pH-6.5) and (pH-7) at 30 °C was 0.066 ± 0.003% and 0.04 ± 0.002%, respectively, while that of hyphae at 37 °C was 0.027 ± 0.002% and 0.008 ± 0.002%, respectively (Table 1). On the other hand, ergosterol content of proline induced hyphae (pH 6.5, proline 2.5 mM) and yeast phase cells (pH 6.5, proline 2.5 mM and LHP 1800 Pa) at 37 °C was found to be 0.013 ± 0.010% and 0.018 ± 0.005%, respectively (Table 1). In general, ergosterol content is more in yeast form cells of *C. albicans* compared to hyphae. However, physical factors like neutral pH and LHP reduce the ergosterol content of yeast phase cells to 0.048 and 0.018%, respectively, compared to 0.061% in control (Table 1).

## 4. Discussion

*Candida albicans* responds to slight changes in environmental conditions and modulates morphophysiology accordingly as a survival strategy [41,42]. It exists in various morphological forms, including unicellular yeast (blastospores), pseudohyphe, hyphae, chlamydospores, and opaque cells [1,7,11,43]. Each of these morphological forms possesses its own characteristic features that exhibit niche specific survival and virulence traits [1,44]. Among these traits, ability to form hyphae is considered as an important prerequisite for *C. albicans* virulence like invasive candidiasis, biolfilm formation etc. [2,45,46]. Hyphae specific cell surface proteins and hydrolytic enzymes were reported to facilitate adhesion, colonization and finally host tissue invasion [47]. Not only virulence, modulated cell morphology as well as cell surface properties enable *C. albicans* cells to respond differentially towards host defense mechanisms as well as anti-fungal agents [48]. Membrane properties determined by fatty acid and sterol composition decides the survival of eukaryotic cells [48,49]. Membrane composition is reported to be modulated in response to various environmental and or nutritional factors to facilitate survival under different microenvironments [48,49]. Modulation of membrane composition (sterols and fatty acids) and thus properties (transport) confer multidrug resistance in *C. albicans* [16,17,18]. Similarly, cell surface chemistry exhibit differential responses towards host cells (target tissues or immune cells) and it is tissue specific [48]. Thus considering its significance in virulence and survival, we have made an attempt to understand modulations in cell surface and membrane properties of *C. albicans* cells (both yeast and hyphae) in response to neutral pH, temperature (37 °C) and LHP (1800 Pa). We have identified modulations in fatty acid content using GC-MS analysis and CSH, adhesion, and ergosterol content in this study.

Temperature is reported to modulate membrane fluidity by changing fatty acid composition [50]. Long-chain unsaturated fatty acids are the common features of the membranes under elevated temperature (37 °C) that increase fluidity while short-chain branched and saturated fatty acids are preferred at 30 °C, as it decreases the membrane fluidity [51,52,53]. Our results also showed the increased percentage of saturated fatty acids, at 30 °C while that of monounsaturated (oleic acid), unsaturated, methyl branched, omega 7-monounsaturated fatty acids etc., at 37 °C indicating increased membrane fluidity at elevated temperature though the variation (abundance of unsaturated fatty acids) is not significant [54]. However this is the first report showing slight modulation in fatty acid composition in response to elevated temperature in addition to morphology. More detailed lipidomic analysis is necessary to understand the significance of temperature induced modulations in fatty acid content in the morphogenesis of *C. albicans*. Similarly, pH is also reported modulating membrane fluidity i.e., fluidity increases with increase in pH (Neutral-alkaline pH) [55]. In our study, neutral pH increased percentage of unsaturated, polyunsaturated fatty acids at 30 °C, indicating more fluid membrane compared to control (6.5 at 30 °C) on the other hand neutral pH at 37 °C further increased membrane fluidity as percentage of monounsaturated (oleic acid), omega-9 fatty acid, omega-5-unsaturatedfatty acid etc., was up-regulated.

Pressure (including hydrostatic pressure) is known to determine the fatty acid composition and thus properties of biological membranes [14]. Lipids are more sensitive to pressure as pressure reduces the acyl chain kinking and thus reported to increase the thickness of lipid bilayers of biological membranes [56]. A gel state transition is promoted by pressure as it causes a tight packing of the membrane to resist the effect of pressure [56,57]. However, the impact of low hydrostatic pressure (considered to be non-significant) on biological membranes is not known. In our study inhibition of hyphae induction intrigued us to understand whether LHP (1800 Pa) has any impact on membrane properties. Increased percentage of medium chain saturated, long chain saturated, methyl-branched saturated fatty acids indicates that LHP reduces membrane fluidity at 37 °C compared to that of without LHP (Supplementary Table S1). However, though monounsaturated and polyunsaturated fatty acids were up-regulated, it was less than that of control without LHP at 37 °C (Supplementary Table S1). Interestingly, our results indicate that LHP inhibits hyphae induction as well as reduce membrane fluidity. Ergosterol content supported our fatty acid data. Ergosterol content was found to be more in yeast form cells grown either at 30 °C (YPD pH 6.5 and pH 7) and 37 °C (YPD (pH 6.5), containing proline (2.5 mM) and LHP (1800 Pa)) compared to hyphal form indicating less fluid membrane (Table 1).

Cell surface chemistry defines the cell wall properties, including cell surface hydrophobicity (CSH) and adhesion etc. [58]. Several factors including physical (pH, temperature, pressure), nutritional and stresses etc., modulating metabolism affects cell surface properties as well [59,60]. Cell surface properties play very important roles in survival and virulence under different microenvironments [1]. CSH determines cell-cell interaction thus adhesion and colonization followed by tissue invasion at different tissue sites with varied microenvironments [61]. In general, CSH and adhesion is directly proportional i.e., adhesion increases with increase in CSH [61,62]. *C. albicans* reported exhibiting significantly more numbers of cell surface adhesins compared to a non-virulent yeast *S. cerevisiae* citing the significance of cell surface molecules in pathogenicity [63]. Our results showed that CSH of yeast phase cells grown at 30 °C (YPD pH 6.5 and 7) is more compared to the hyphae induced at 37 °C (YPD pH 6.5 and 7). However, yeast phase cells grown in presence LHP at 37 °C were less hydrophobic than hyphal form cells grown in absence of LHP at 37 °C. CSH and adhesion showed positive correlation, i.e., adhesion increased with increase in CSH [22,23]. It indicates that irrespective of the morphological form (yeast or hyphae), cell surface properties are modulated significantly in response to micro-environments.

## 5. Conclusions

Our results showed that physical factors, including temperature (37 °C), neutral pH, and LHP (1800Pa) modulate membrane fluidity by changing fatty acid composition and ergosterol content. Membranes of hyphae induced by temperature alone and in combination with neutral pH and/or proline are more fluid compared to the yeast phase cells grown at pH 6.5 and 7 at 30 °C. In addition to this, CSH and adhesion were found to be different in the cells grown under different physical factors. It indicates that CSH and adhesion are dynamic properties determined largely by the microenvironment rather than the morphological forms citing the significance of variation in niche-specific virulent traits.

## Figures and Tables

**Table 1:** Effect of temperature, pH, and low hydrostatic pressure (LHP) on hyphae induction, cell surface hydrophobicity, adhesion, and ergosterol content in *C. albicans* (ATCC 10231).

Growth Medium	Morphology, Cell Surface Hydrophobicity, Adhesion and Ergosterol Content	% ± S.D.	
		30 °C	37 °C
YPD broth (pH 6.5)	Hyphae	08.0 ± 1.0	65.0 ± 2.51
	CSH	26.36 ± 1.57	22.87 ± 0.50
	Adhesion	56.0 ± 3.0	23.67 ± 2.08
	Ergosterol	0.066 ± 0.003	0.027 ± 0.002
YPD broth (pH 7.0)	Hyphae	10.0 ± 1.0	85.0 ± 2.0
	CSH	11.71 ± 0.56	6.6 ± 0.48
	Adhesion	68.0 ± 4.0	30.6 ± 1.52
	Ergosterol	0.04 ± 0.002	0.008 ± 0.002
YPD broth (pH 6.5, Proline2.5 mM)	Hyphae	n/a.	85.0 ± 1.52
	CSH		10.3 ± 0.39
	Adhesion		51.0 ± 1.0
	Ergosterol		0.013 ± 0.010
YPD broth (pH 6.5, Proline2.5 mM, LHP-1800 Pa)	Hyphae	n/a.	10.0 ± 0.57
	CSH		5.97 ± 0.07
	Adhesion		20.66 ± 1.52
	Ergosterol		0.018 ± 0.005

## Supplementary Materials

The following are available online at http://www.mdpi.com/2309-608X/4/2/47/s1, Figure S1. Identification of Fatty acid content in *Candida albicans* cells grown under Temperature at 30 °C; Figure S2. Identification of Fatty acid content in *Candida albicans* cells grown under Temperature at 37 °C; Figure S3. Identification of Fatty acid content in *Candida albicans* cells grown under pH 7 at 30 °C; Figure S4. Identification of Fatty acid content in *Candida albicans* cells grown under pH 7 at 37 °C; Figure S5. Identification of Fatty acid content in *Candida albicans* cells grown under LHP (control 100 Pa); Figure S6. Identification of Fatty acid content in *Candida albicans* cells grown under LHP at 37 °C (1800 Pa); Table S1. Modulation of fatty acid content in *Candida albicans* cells (ATCC 10231) grown under different environmental condition.
