# Supplementary material for: Microenvironment Responsive Modulations in the Fatty Acid Content, Cell Surface Hydrophobicity, and Adhesion of Candida albicans Cells
Source: J Fungi (Basel). 2018 Apr 6;4(2):47. doi: 10.3390/jof4020047 (PMC6024300; doi:10.3390/jof4020047)
Supplement: Supplementary file 1 [file jof-04-00047-s001.pdf]

Supplementary data

Figure S1. Identification of Fatty acid content in *Candida albicans* cells grown under Temperature at 30°C

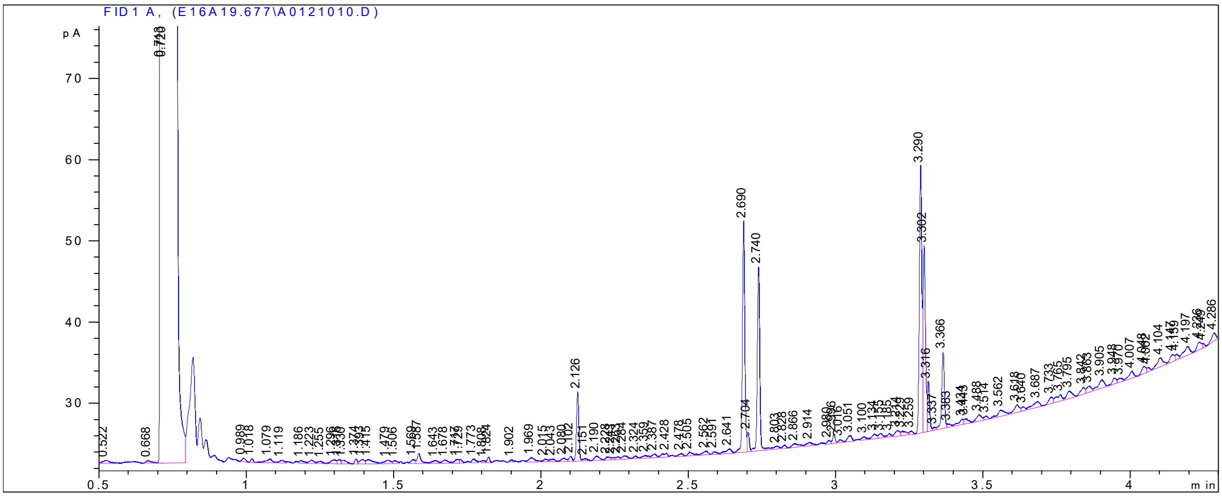

X axis – Retention time

Y axis – Mass

Figure S2. Identification of Fatty acid content in *Candida albicans* cells grown under Temperature at 37°C

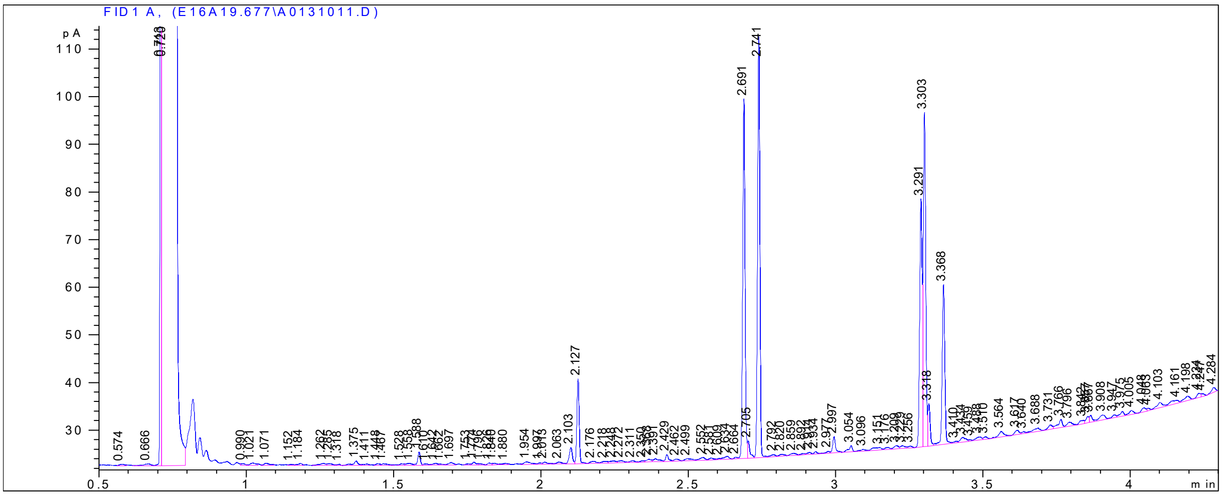

X axis – Retention time

Y axis – Mass

Figure S3. Identification of Fatty acid content in *Candida albicans* cells grown under pH 7 at 30°C

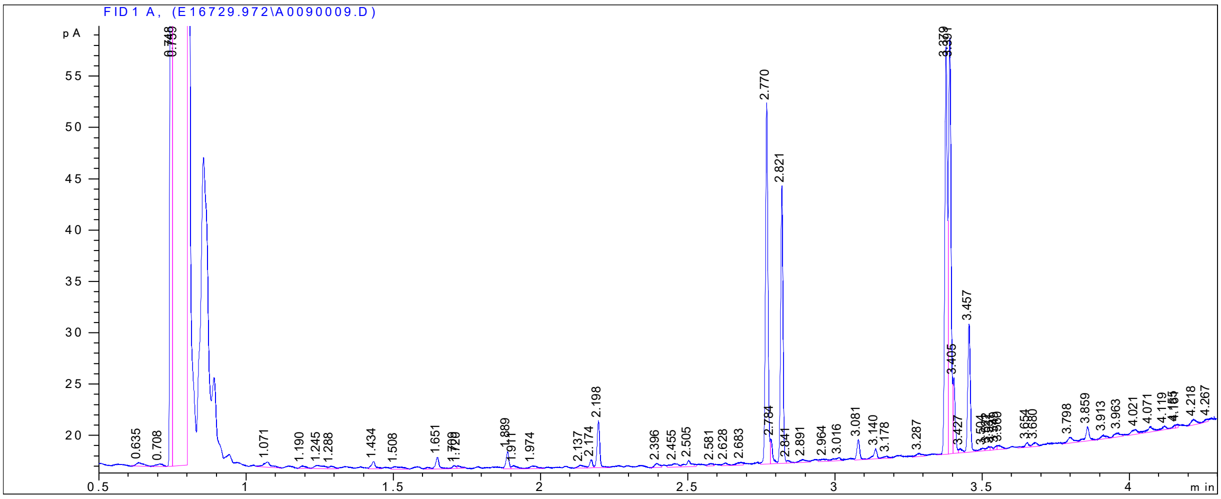

X axis – Retention time

Y axis – Mass

Figure S4. Identification of Fatty acid content in *Candida albicans* cells grown under pH 7 at 37°C

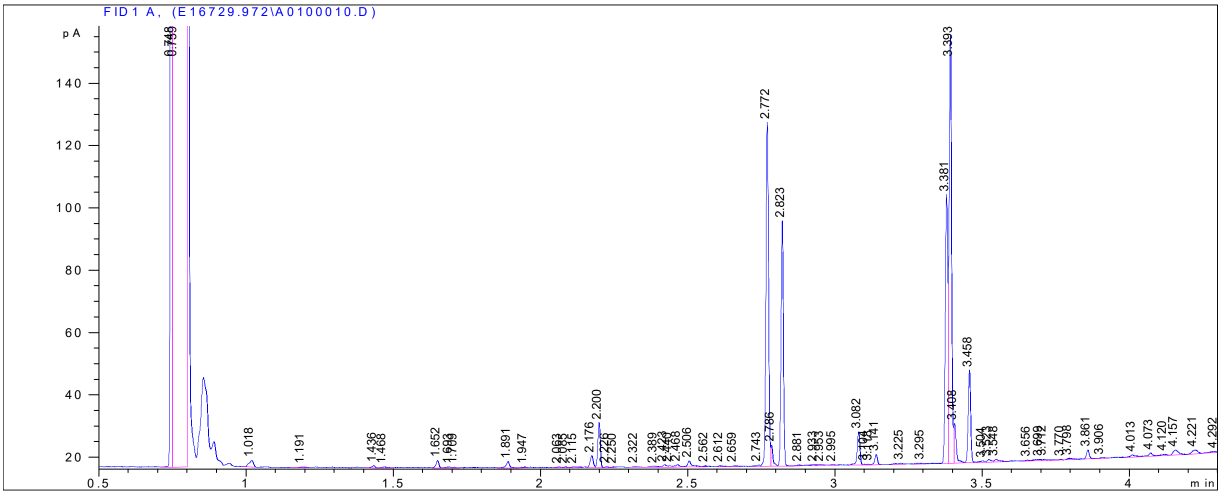

X axis – Retention time

Y axis – Mass

Figure S5. Identification of Fatty acid content in *Candida albicans* cells grown under LHP (control 100 Pa)

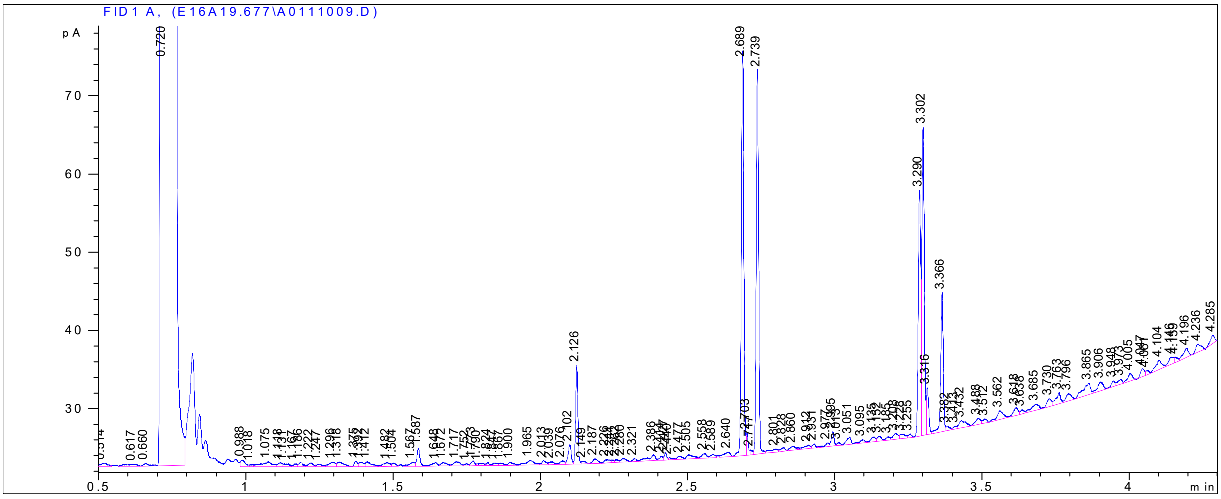

X axis – Retention time

Y axis – Mass

Figure S6. Identification of Fatty acid content in *Candida albicans* cells grown under LHP at 37°C (1800 Pa)

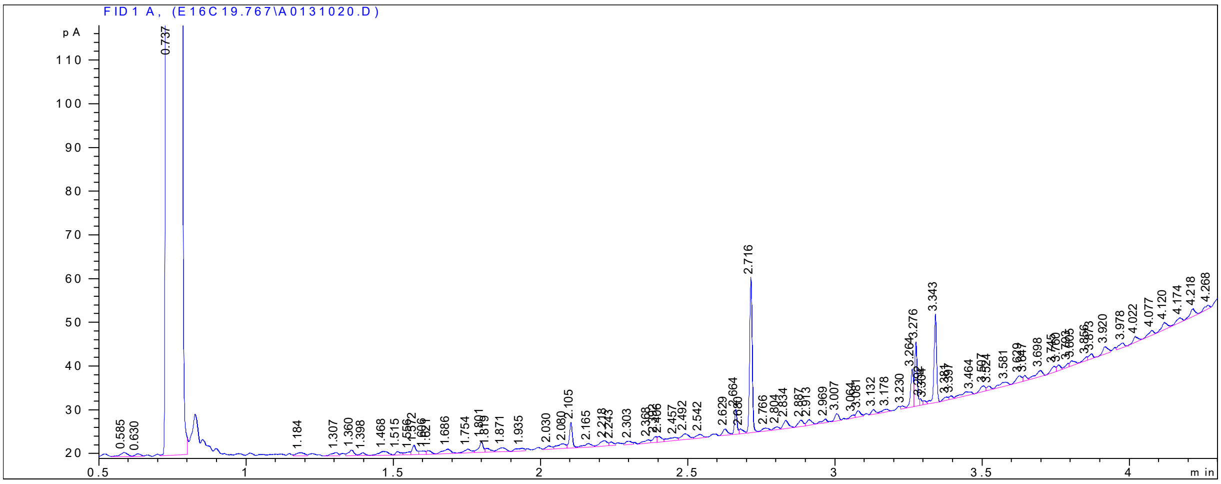

X axis – Retention time

Y axis – Mass

Table S1. Modulation of fatty acid content in *Candida albicans* cells (ATCC 10231) grown under different environmental condition.

Sr. No.	Fatty acid Name	Types of fatty acid	Fatty acid (%)					
			Temperature		Neutral pH		LHP at 37˚C (Pa)	
			30˚C	37˚C	30˚C	37˚C	100	1800
1	Dodecanoic acid (12:0)	Medium chain Saturated	0.98	0.91	1.13	0.58	1.47	2.63
2	(9Z)-9-Tetradecenoic acid(14:1 w5c)	Omega-5 unsaturated	0.90	-	0.52	0.97	-	-
3	Tetradecanoic acid(14:0)	Long chain Saturated	9.47	4.74	2.74	2.81	4.46	4.63
4	13-Methyltetradecanoic acid(15:0 iso)	Long chain saturated	0.58	-	0.43	0.19	0.33	-
5	Pentadecanoic acid(15:0)	Long chain Saturated	-	-	-	-	-	-
6	2-Hydroxytetradecanoic acid(14:0 2OH)	Hydroxy saturated	1.10	-	0.26	-	0.49	-
7	Hexadecanoic acid(16:0)	Long chain Saturated	25.23	22.23	14.20	14.82	20.40	23.32
8	Heptadecanoic acid(17:0)	Long chain Saturated	-	0.53	0.76	0.69	-	-
9	(9Z)-9-Octadecenoic acid(18:1 w9c)	Monounsaturated	-	18.12	22.91	26.79	20.91	9.59
10	Octadecanoic acid(18:0)	Long chain Saturated	-	9.13	6.90	5.76	12.43	13.60
11	17-Methyloctadecanoic acid(19:0 iso)	Methyl branched Saturated	-	0.68	0.40	0.10	1.55	-
12	18-Methylnonadecanoic acid(20:0 iso)	Methyl branched Saturated	-	0.42	0.52	-	1.68	-
13	Icosanoic acid(20:0)	Long chain saturated	-	0.42	0.75	0.29	-	-
14	2-Hydroxydecanoic acid(10:0 2OH)	Short chain Saturated	-	0.22	-	0.20	-	0.90
15	12-Methyltetradecanoic acid(15:0 anteiso)	Branched fatty acid, methyl branched, saturated fatty acid and derivatives.	-	0.14	-	0.26	-	-
16	Decanoic acid(10:0)	Medium chain Saturated	0.98	-	-	-	0.68	1.96
17	9-Methyldecanoic acid(11:0 iso)	Methyl branched Saturated	1.50	-	-	-	-	-
18	8-Methyldecanoic acid(11:0 anteiso)	Methyl branched Saturated	0.87	0.13	-	-	-	1.59
19	10-Methylundecanoic acid(12:0 iso)	Methyl `Branched Saturated	0.89	-	-	-	0.55	-
20	10-Methyldodecanoic acid(13:0 anteiso)	Methyl Branched Saturated	1.04	0.29	-	-	0.90	1.77
21	2-Hydroxydodecanoic acid(12:0 2OH)	long chain hydroxy	0.43	-	-	-	-	-
22	15:1 iso F	long chain saturated	0.64	-	-	-	-	-
23	3-Hydroxy-12-Methyltridecanoic acid(14:0 iso 3OH)	Hydroxy saturated	0.74	-	-	-	0.66	-

24	(9Z)-9-Hexadecen-1-ol(16:1 w7c alcohol)	unsaturated fatty alcohol	1.23	-	-	-	0.74	-
25	14:0 3OH	long chain saturated	0.66	-	-	-	0.43	-
26	unknown 15.669	---	-	-	-	-	-	-
27	(9Z)-9-Hexadecenoic acid (16:1 w7c)	Omega-7 monounsaturated	2.37	19.7 8	-	-	17.58	-
28	15:0 2OH	long chain saturated	0.64	0.24	-	-	0.42	-
29	15:0 3OH	long chain saturated	1.22	-	-	-	-	-
30	14-Methylhexadecanoic acid(17:0 anteiso)	Methyl Branched Saturated	0.64	0.17	-	-	0.48	-
31	18:1 iso H	long chain saturated	1.39	0.33	-	-	0.79	-
32	16:0 3OH	long chain saturated	1.20	0.28	-	-	0.72	--
33	16-Methylheptadecanoic acid(18:0 iso)	Methyl branched	0.72	0.20	-	-	0.35	0.65
34	(9Z,12Z)-9,12-Octadecadienoic acid (18:2 w6c)	Polyunsaturated Omega- 6	36.3 0	13.4 5	-	-	-	5.76
35	17:0 iso 3OH	long chain saturated	1.46	0.53	-	-	1.42	-
36	(10Z)-10-Nonadecenoic acid (19:1 w9c)	Long chain saturated	2.07	-	-	-	-	2.16
37	(12Z)-12-Nonadecenoic acid (19:1 w7c)	Long chain saturated	0.76	0.12	-	-	0.43	-
38	(11Z,14Z)-11,14-Icosadienoic acid(20:2 w6,9c)	Polyunsaturated	2.65	-	-	-	-	-
39	14:0 3OH	Long chain saturated	0.66	-	-	-	0.43	-
40	(11Z)-11-Octadecenoic acid (18:1 w7c)	omega-7unsaturarted	-	2.19	-	-	3.57	0.94
41	Undecanoic acid(11:0)	Medium chain saturated	-	0.69	-	-	-	2.15
42	3-Hydroxydecanoic acid(10:0 3OH)	Medium chain saturated	-	0.30	-	-	-	-
43	9-Methylundecanoic acid(12:0 anteiso)	Methyl Branched Saturated	-	0.18	-	-	0.28	1.11
44	11:0 2OH	Medium chain saturated	-	0.23	-	-	-	1.15
45	11-Methyldodecanoic acid(13:0 iso)	Methyl Branched Saturated	-	0.13	-	-	-	-
46	1-Hexadecanol(16:0 N alcohol)	Long chain Fatty alcohol	-	0.08	-	-	-	-
47	15:0 iso 3OH	long chain saturated	-	0.22	-	-	-	1.08
48	17:0 2OH	Long chain saturated	-	0.09	-	-	-	-
49	18:0 2OH	Long chain saturated	-	0.80	-	-	0.61	-
50	Tridecanoic acid(13:0)	Long chain Saturated	-	-	0.42	-	0.29	-
51	Unidentified	---	-	-	18.9 0	22.00	-	-
52	Unidentified	---	-	-	1.28	1.13	-	-
53	Unidentified	---	-	-	1.13	-	-	-
54	(9Z)-9-Heptadecenoic acid(17:1 w8c)	Long chain saturated	-	-	1.22	2.25	-	-
55	Unidentified	---	-	-	20.7 5	15.71	-	-

56	Unidentified	---	-	-	3.50	2.43	-	-
57	Unidentified	---	-	-	0.32	-	-	-
58	Unidentified	---	-	-	0.17	0.34	-	-
58	Unidentified	---	-	-	1.12	0.64	-	-
59	(5Z,8Z,11Z,14Z)-5,8,11,14-Icosatetraenoic acid(20:4 w6c)	Polyunsaturated	--	-	0.43	-	-	-
60	Unidentfied	---	-	-	20.18	23.13	-	-
61	Unidentfied	---	-	-	20.75	20.75	-	-
62	Unidentfied	---	-	-	3.82	2.43	-	-
63	Unidentfied	---	-	-	0.36	-	-	-
64	11-Methyltridecanoic acid(14:0 anteiso)	Long chain Saturated	-	-	-	0.18	-	1.24
65	(9Z)-9-Pentadecenoic acid(15:1 w6c)	Long chain Saturated	-	-	-	0.28	-	1.32
66	Unidentfied	---	-	-	-	0.12	-	-
67	Unidentfied	---	-	-	-	0.14	-	-
68	(7Z)-13-Methyl-7-Hexadecenoic acid(17:1 anteiso w9c)	Long chain Saturated	-	-	-	0.11	0.56	1.86
69	(11Z)-11-Heptadecenoic acid(17:1 w6c)	Long chain Saturated	-	-	-	0.05	0.31	-
70	(12Z)-12-Heptadecenoic acid(17:1 w5c)	Long chain Saturated	-	-	-	0.06	-	1.69
71	Unidentfied	---	-	-	-	0.16	-	-
72	Nonadecanoic(19:0)	Long chain saturated	-	-	-	0.11	-	1.05
73	(11Z)-11-Icosenoic acid(20:1 w9c)	Monounsaturated Omega-9	-	-	-	0.29	-	-
74	Unidentfied	---	-	-	-	0.22	-	-
75	10:0 iso	Medium chain saturated	-	-	-	-	0.51	-
76	12:1 3OH	Medium chain saturated	-	-	-	-	0.28	-
77	15:1 iso F	Long chain saturated	-	-	-	-	0.70	-
78	13:0 3OH	Medium chain saturated	-	-	-	-	0.65	-
79	(11Z)-11-Hexadecenoic acid(16:1 w5c)	Long chain saturated	-	-	-	-	0.29	-
80	10-Methyloctadecanoic acid(18:0 10-methyl, TBSA)	Methyl branched saturated	-	-	-	-	1.14	-
81	12:1 at 11-12	Medium chain saturated	-	-	-	-	-	1.02
82	3-Hydroxy-9-Methyldecanoic acid(11:0 iso 3OH)	Branched chain hydroxyl	-	-	-	-	-	1.31
83	14:1 iso E	Long chain saturated	-	-	-	-	-	1.51
84	15:1 iso G	Long chain saturated	-	-	-	-	-	1.06
85	15-Methylhexadecanoic acid(17:0 iso)	Methyl Branched Saturated	-	-	-	-	-	1.64
86	16:1 2OH	Long chain saturated	-	-	-	-	-	0.69
87	16:0 iso 3OH	Long chain saturated	-	-	-	-	-	1.63

88	19:0 cyclo w8c	Long chain saturated	-	-	-	-	-	2.04
89	(5Z,8Z,11Z,14Z)-5,8,11,14-Icosatetraenoic acid(20:4 w6,9,12,15c)	Polyunsaturated	-	-	-	-	-	0.73
90	(11Z,14Z)-11,14-Icosadienoic acid(20:2 w6,9c)	Polyunsaturated	-	-	-	-	-	1.20
